# Evaluation of the Significance of Pretreatment Liver Biopsy and Baseline Mental Health Disorder Diagnosis on Hepatitis C Treatment Completion Rates at a Veterans Affairs Medical Center

**DOI:** 10.1155/2013/653976

**Published:** 2013-05-16

**Authors:** Joseph Kluck, Rose M. O'Flynn, David E. Kaplan, Kyong-Mi Chang

**Affiliations:** ^1^Department of Pharmacy, Philadelphia Veterans Affairs Medical Center, 3900 Woodland Avenue, Philadelphia, PA 19104, USA; ^2^Department of Pharmacy, Hospital of the University of Pennsylvania, 3400 Spruce Street, Philadelphia, PA 19104, USA; ^3^Gastroenterology Section, Philadelphia Veterans Affairs Medical Center, 3900 Woodland Avenue, Philadelphia, PA 19104, USA; ^4^Division of Gastroenterology, University of Pennsylvania Perelman School of Medicine, 421 Curie Boulevard, 9th Floor, Philadelphia, PA 19104, USA

## Abstract

*Objectives*. This study was performed to define the overall treatment response rates and treatment completion rates among the population of Hepatitis C infected patients at an urban VA Medical Center. Additionally, we examined whether pretreatment liver biopsy is a positive predictor for treatment completion and if the presence of mental health disorders is a negative predictor for treatment completion. *Methods*. Retrospective chart review was performed on the 375 patients that were treated for HCV and met the study inclusion parameters between January 1, 2003 and April 1, 2008 at our institution. Clinical data was obtained from the computerized patient record system and was analyzed for respective parameters. *Results*. Sustained virological response was achieved in 116 (31%) patients. 169 (45%) patients completed a full treatment course. Also, 44% of patients who received a pre-treatment liver biopsy completed treatment versus 46% completion rates for patients who did not receive a pretreatment liver biopsy. Baseline ICD9 diagnosis of a mental health disorder was not associated with higher treatment discontinuation rates. *Conclusions*. In conclusion, pretreatment liver biopsy was not a positive predictor for treatment completion, and the presence of mental health disorders was not a negative predictor for treatment completion.

## 1. Background 

Hepatitis C virus (HCV) is a highly persistent, hepatotropic RNA virus that causes chronic necroinflammatory liver disease [1]. HCV seroprevalence is 2.2% in the world, 1.6% in the USA and as high as 15-16% in select Veterans Affairs Medical Centers (VAMC) [2–6]. Comorbid factors common among the US veteran population, such as advanced age, obesity, HIV coinfection, immunosuppression, and alcohol intake, are associated with accelerated liver disease and progression to cirrhosis in chronically HCV-infected patients [7–9]. HCV-associated cirrhosis (occurring in 20–30% of HCV-infected individuals) results in increased morbidity and mortality due to end stage liver failure and hepatocellular carcinoma (HCC) that may warrant liver transplantation [10–12]. Thus, the goal of HCV therapy is to clear HCV RNA in an effort to prevent or delay liver-related death and/or complications [7, 9].

The desired objective outcome of HCV-directed therapy is a sustained virologic response (SVR), which is defined as an undetectable HCV viral load (VL) 24 weeks after therapy completion and denotes a cure of the infection [7]. Several variables are known to predict the likelihood of SVR with pegylated interferon and ribavirin treatment. Other than HCV genotype, which has the strongest impact on SVR rates, favorable treatment response is associated with pretreatment HCV VL below 600,000 IU/mL, female gender, age less than 40 years old, race/ethnicity other than black/African American, body weight less than 75 kg, absence of insulin resistance, elevated ALT levels, and absence of bridging fibrosis or cirrhosis [7]. In the VA system, the characteristics associated with a favorable treatment response are underrepresented, as a high proportion of patients are males, greater than 50 years of age, of African American background, cirrhotic, have high HCV viral loads, and weigh more than 75 kg [13]. In addition, many HCV-infected patients in the VA system display other comorbidities that limit HCV treatment tolerability and efficacy, such as HIV coinfection, poorly controlled diabetes, morbid obesity, and psychiatric disorders including depression, posttraumatic stress disorder, and schizophrenia, or recent substance abuse [13–15]. Since these comorbidities are not well represented in most large randomized clinical trials for HCV therapy [16–20], it is difficult to extrapolate the findings in the published literature to the veteran population [13].

At the time of our study protocol development and throughout the evaluation period, the accepted standard treatment of HCV involved combination pegylated interferon plus ribavirin for a total of 24 weeks for HCV genotypes 2 and 3, or 48 weeks for HCV genotypes 1 and 4 [7, 21–23]. SVR has been shown to occur more often in patients that complete a full course of treatment compared to patients that discontinue treatment early [13, 22, 24].

The main goals of this study were to define the overall treatment response and treatment completion rates among our population of HCV-infected veterans at the Philadelphia VA Medical Center. Additionally, we examined how completion rates were influenced by psychological factors. Two different concepts were proposed for evaluating this domain. The first concept aimed to determine whether or not patients that began therapy within one year of receiving a liver biopsy would be more likely to complete treatment. The second focused on evaluating if the diagnosis of a mental health disorder at baseline affected HCV therapy completion rates.

We hypothesized that patients may be more likely to complete therapy if they undergo staging of liver disease by means of biopsy. Does having a biopsy, which is an invasive procedure, psychologically motivate patients to complete therapy [25]? A patient's willingness to undergo a liver biopsy may indicate that the patient is mentally prepared to initiate and complete the extensive treatment for HCV. One might also predict that patients who do not receive liver biopsy might be less aware of their disease severity and may be less likely to see treatment to completion.

HCV-drug therapy has been widely associated with causing psychiatric side effects, often leading to early discontinuation of treatment [13]. To determine if the presence of mental health disorders is a negative predictor for treatment completion, we assessed whether the number of mental health disorders at baseline affected rates of HCV therapy completion. We also evaluated the rates of therapy discontinuation, specifically due to psychiatric adverse effects, amongst patients with and without a mental health disorder.

## 2. Patients and Methods

### 2.1. Study Population

HCV-infected veterans who were initiated on pegylated interferon alpha- (PEG-IFN-) based therapy at the Philadelphia VAMC between January 1, 2003 and April 1, 2008 were retrospectively identified by using the pharmacy database and examined for baseline demographic, clinical, and HCV treatment information using the Computerized Patient Record System (CPRS). Given the evolving nature of HCV therapy, we excluded patients whose HCV treatment prescriptions were filled within the study period but had initiated treatment before January 1, 2003. We also excluded patients whose prescriptions were filled within the desired timeline but were documented as either having received fewer than four weeks of antiviral therapy, or having never initiated therapy. This study protocol was reviewed and approved by the Institutional Review Board at the Philadelphia Veterans Affairs Medical Center (VAMC).

If a patient received multiple courses of HCV treatment, information on the most recent course of therapy was collected since retreatment would only occur if the patient failed therapy due to intolerance or lack of efficacy.

### 2.2. Clinical Data Collection from CPRS

Baseline patient information included age, race, gender, weight, HCV genotype, liver transplantation status, hepatic fibrosis (by liver biopsy or Fibrosure, when available), and significant comorbid illnesses recorded at baseline before HCV therapy. Race was obtained by chart documentation based on patient self-report. Mental health disorder diagnoses and diabetes diagnosis were determined by ICD9 codes, progress notes, and/or pharmacy medication records. HIV status was determined by laboratory screening when available. Baseline laboratory data was recorded as the most recent value documented within 12 months prior to the first dose of interferon and ribavirin. Concomitant alcohol or substance abuse was documented based on progress notes.

Initial treatment regimen and on-treatment alterations were retrieved from pharmacy records and/or in progress notes. Stop dates for treatment were defined as 4 weeks after the last prescription fill date for interferon or ribavirin, unless specifically stated otherwise in progress notes. The number of treatment courses was determined using pharmacy records, unless the number of treatment courses was specifically mentioned in progress notes since some patients may have previously been treated outside of the VA system. Completion rates were determined by the occurrence of treatment ending earlier than the specified treatment length, as determined by individual viral genotype and by provider comments in progress notes. Reasons for early termination of treatment were recorded if specifically stated in progress notes.

### 2.3. Definition of HCV Treatment Responses

HCV treatment responses were based on Roche Cobas Amplicor Taqman HCV RNA assay used in the clinical laboratory. The starting date of treatment was used as a marker to assess the monitoring of viral load for rapid virological response (RVR), early virological response (EVR), end-of-treatment response (ETR), and sustained virological response (SVR) per the definition of HCV diagnosis, management, and treatment guidelines [7]. Treatment failure was defined as less than a 2 log decrease in HCV RNA at week 12 or if HCV RNA was greater than 0 at week 24 [7].

Laboratory data, pharmacy records, and progress notes were used to retrieve data for adverse effects. Anemia, neutropenia, and thrombocytopenia occurring during the studied course of treatment were recorded. Pharmacy records were used to determine if growth factors such as erythropoietin or filgrastim were utilized to treat respective adverse effects.

## 3. Results

### 3.1. Patient Selection and Characteristics

Between January 1, 2003 and April 1, 2008 at the PVAMC, a total of 463 patients were identified as receiving prescriptions for pegylated-interferon and ribavirin. Eighty-eight of the 463 subjects were excluded for the following reasons: 24 (27.3%) initiated therapy before January 2003; 36 (40.9%) filled their first prescription but reported never initiating therapy; 16 (18.2%) stopped therapy within 4 weeks due to adverse effects; 12 (13.6%) filled the first prescription but had fewer than 4 weeks of followup documented. Thus, a total of 375 HCV-treated patients were included in the study.

### 3.2. Baseline Results

Among the 375 total subjects, 98% were male, 51% were of black racial background, and 80% had genotype 1 or 4 infections (Table 1). Median age among included subjects was 53 years (range 27–77). Median BMI was 29.2 kg/m^2^ (95% CI 18.7–59.5) where 37% of patients were overweight, 40% of patients were obese, and 4% of patients were morbidly obese. Median pretreatment HCV viral titers were 1,350,000 IU/mL (range 344–25,400,000). Diabetes was common in the cohort, affecting 97 (26%) patients. HIV coinfection was present in 35 (9%) while 87 (23%) did not have a documented HIV assessment. A total of 252 (67%) patients received only one course of HCV treatment, as compared to 33% of patients who received two or more courses of therapy. Concomitant substance abuse was reported by 11 (3%) patients while 30 (8%) patients reported use of alcohol during treatment.

Mental health disorders were common, with 224 (59.7%) patients having at least one mental health disorder (Table 2). Subjects were found to commonly have multiple mental health disorders with depressive disorders, posttraumatic stress disorders, and anxiety disorders, most frequently represented.

### 3.3. Sustained Virological Response (SVR) Relative to HCV Genotype, Ethnicity, and Treatment Completion

SVR was achieved in 116/375 (31%) patients. The remaining patients were either well-documented nonresponders (187 (50%)) or lacked documentation of response (72 (19%)). As expected, SVR rate was lower for patients infected with genotypes 1 and 4 than genotypes 2 and 3 (23% versus 59%, *P* < 0.0001). However, SVR rates did not differ significantly by ethnicities in patients with HCV genotypes 1 and 4 (21.1% white versus 23.6% black, *P* = 0.7580) or HCV genotypes 2 and 3 (66.7% white versus 37.5% black, *P* = 0.2317). Overall, 169 (45%) patients completed a full treatment course, respective for specific HCV genotype. Median duration of therapy among genotypes 1 and 4 was 48.3 weeks for patients that achieved SVR and 30.1 weeks for patients that did not respond to therapy. Median duration of therapy among genotypes 2 and 3 was 24.7 weeks for patients that achieved SVR and 20.7 weeks for patients that did not achieve SVR.

### 3.4. Is Pretreatment Liver Biopsy a Positive Predictor of Treatment Completion?

Eighty-five (23%) patients received a liver biopsy within one year of starting HCV treatment. Of these 85 patients, 37 (44%) completed the full course of therapy. Of the 290 included patients that did not receive a liver biopsy within one year of starting HCV treatment, 132 (46%) completed therapy (*χ* ^2^, *P* = 0.81). Therefore, there was no statistical difference found regarding treatment completion rates in patients who received a biopsy when compared to patients who did not receive a biopsy prior to initiation of treatment.

### 3.5. Does Having a Mental Health Disorder Affect Completion of Antiviral Therapy?

Early discontinuation of HCV therapy was common with more than half of the patients (206/375 (55%)) stopping therapy prior to completion of the full course. The most common reasons for early discontinuation were (1) medication-related side effects (46%), (2) virological failure (32%), and (3) loss to followup (15%). To determine the impact of mental health disorders on completion rates, we assessed treatment completion rates across three groups: patients without a mental health disorder at baseline, patients with one mental health disorder at baseline, and patients with two or more mental health disorders at baseline (Table 3). No statistical difference was found for completion rates for these groups (*P* = 0.33).

Additionally, we assessed early termination of treatment rates involving psychiatric related adverse drug effects. Of the 94 patients that stopped treatment early due to any medication related side effects, 59 (63%) had at least one documented mental health disorder prior to the start of HCV treatment, whereas 35 (37%) had no history of a mental health diagnosis. Twenty-four (41%) of these 59 patients stopped treatment early specifically due to psychiatric related adverse drug effects, compared with 10 of these 35 (29%) who did not have a mental health disorder at the start of HCV treatment (*P* = 0.27). Thus, the baseline diagnosis of a mental health disorder was not associated with higher treatment discontinuation rates in our patients.

## 4. Discussion

Our results indicate that there is no difference in rates of completion when considering biopsy status within one year of starting HCV treatment. While biopsy status had no impact on treatment completion rates, it is likely that biopsy status correlated with treatment uptake as the biopsy rate of this cohort (23%) was significantly greater than the rate of liver biopsy in the untreated chronic hepatitis C population at our center (3.8%). We did not collect provider level information in this analysis, but it is also highly likely that provider practice style might strongly influence patient persistence during therapy.

In theory, patients with mental health disorders, at baseline, would seem less likely to tolerate and complete a full course of HCV treatment, as they may be more sensitive to the psychiatric adverse effect profile of interferon-alpha. To assess the impact of a mental health disorder at baseline on HCV therapy completion rates, we assessed whether the number of mental health disorders at baseline affected rates of HCV therapy completion and rates of therapy discontinuation related to psychiatric adverse effects for patients with relative to those without mental health disorders. Pegylated interferon and ribavirin can cause symptoms of anxiety and depression at an incidence of 20–30% [26–28]. Some studies have shown that almost 50% of patients undergoing antiviral treatment can experience symptoms of anxiety, depression, or irritability [29]. Additional studies have shown that interferon induced depression significantly contributes to early discontinuation of treatment and subsequently lower incidence of SVR [30–32]. Interestingly, our study showed that the rates of HCV therapy discontinuation due to psychiatric-related adverse drug effects were similar between patients with a mental health disorder at baseline and with no mental health disorder at baseline.

Since complete information on patients lost to follow-up was not available, it is possible that psychiatric adverse effects contributed to these discontinuations. Therefore, we determined that it was necessary to evaluate overall completion rates for patients with mental health disorders at baseline. We also intended to determine if there was any difference in completion rates among patients with different numbers of mental health diagnoses at baseline. Liu et al. evaluated a random sample of patients treated in the HCV clinic and noted that patients with a diagnosis of depression at baseline had lower rates of completion than patients without preexisting depression [33]. Previous studies have also shown that patients with schizophrenia have similar completion rates as patients without schizophrenia [34]. Our study revealed that patients with one or more mental health disorders at baseline had similar rates of completion when compared to patients without any diagnosed mental health disorder.

Our study found that discontinuation of treatment due to psychiatric related adverse drug effects was similar among all patients, regardless of mental health disorder diagnosis at baseline. Additionally, patients with any number of mental health disorders at baseline have similar rates of completion as patients without a mental health disorder. With this being said, it is difficult to ignore the role that mental health plays with HCV treatment. Mental health disorders should not necessarily be seen as a barrier to HCV treatment. It may be more important for all patients, regardless of psychiatric history, to have close monitoring and individualization of their care, as supported by a recent study and treatment guidelines [7, 35].

Several quality improvement measures were identified for our HCV clinic, as a result of this evaluation. This study did indicate that it is necessary to standardize the monitoring and followup of HCV-treated patients at our institution, so that 100% of patients are consistently assessed for adverse drug effects, HIV-status, reason for early discontinuation of treatment, and viral load at appropriate time points.

There were several potential limitations of our study. Our study contained many variables over a large span in time. Specific changes in HCV therapy identified between the study dates included adherence to checking viral load, aggressiveness in management of adverse effects, practice styles, treatment guidelines, and the actual viral assay. In addition, data was not collected regarding patients' mental health stability or use of psychiatric medications at baseline. This potential for bias was known prior to preparation of the study, as this was a retrospective analysis. Some data was self-reported by patients, such as concomitant alcohol and substance abuse during the treatment course, and this information is potentially biased to underreporting. Another limitation was also attributed to the study design. Incomplete data was occasionally observed, due to lack of documentation and patients lost to follow-up.

In conclusion, in this retrospective analysis, liver biopsy within one year of starting HCV therapy was not associated with increased rates of therapy completion. Similar completion rates were found between patients without a mental health disorder at baseline, with one mental health disorder prior to treatment, and with two or more mental health disorders at baseline. Treatment discontinuation due to psychiatric-related adverse drug effects was found to be similar among patients, regardless of psychiatric history.

## Figures and Tables

**Table 1:** Baseline characteristics.

Variables	Cohort (*n* = 375)
Gender	
Male, *n* (%)	367 (98%)
Female, *n* (%)	8 (2%)
Race/ethnicity	
Black, *n* (%)	192 (52%)
White, *n* (%)	141 (38%)
Unknown, *n* (%)	22 (6%)
Other, *n* (%)	20 (5%)
Genotype	
1 and 4, *n* (%)	301 (80%)
2 and 3, *n* (%)	58 (15%)
Not documented, *n* (%)	12 (3%)
Mixed, *n* (%)	4 (1%)
Age (years), median (range)	53 (27–77)
BMI (kg/m^2^)	29.2 (18.7–59.5)
Obese (BMI 30 to 39.9), *n* (%)	149 (40%)
Overweight (BMI 25 to 29.9), *n* (%)	137 (37%)
Morbidly obese (BMI ≥ 40), *n* (%)	15 (4%)
HCV VL RNA (IU/mL), median (range)	1,350,000 (344–25,400,000)
Diabetes diagnosis, *n* (%)	97 (26%)
HIV diagnosis	
No, *n* (%)	253 (68%)
Not assessed or not documented, *n* (%)	87 (23%)
Yes, *n* (%)	35 (9%)
Course of therapy	
1st, *n* (%)	252 (67%)
2nd, *n* (%)	92 (25%)
3rd or more, *n* (%)	31 (8%)
Concomitant substance abuse, *n* (%)	11 (3%)
Concomitant alcohol use, *n* (%)	30 (8%)
Alb (g/dL), median (range)	4.2 (2.7–5.3)
Hgb (g/dL), median (range)	14.6 (9.8–18.2)
INR, median (range)	1.0 (0.86–1.78)
PLT (THO/uL), median (range)	197.5 (42–439)
SCr (mg/dL), median (range)	1.0 (0.6–3.5)
Tbili (mg/dL), median (range)	0.8 (0.1–2.9)
WBC (THO/uL), median (range)	6.2 (2.2–14.9)

**Table 2:** Frequency of specific mental health disorder.

Mental health disorder, ICD-9 diagnosis	Frequency, *n*	%
Depressive disorder	125	42
Posttraumatic stress disorder	90	30
Anxiety disorder (not specified)/panic disorder/social phobia/obsessive compulsive disorder	37	12
Schizophrenia/psychosis/thought disorder	24	8
Bipolar disorder	13	4
Mood disorder (not specified)	7	2
Cognitive disorder/organic brain disorder	4	1
Personality disorder	4	1

**Table 3:** Summary of completion rate by the number of mental health disorders.

Mental health disorder at baseline	*n* (%), patients completed HCV treatment course**
0	62/151 (41%)
1	70/152 (46%)
2 or more	37/72 (51%)

**Chi-square analysis shows that no statistical difference was found for completion rates across these strata (*P* = 0.33).

## Abbreviations

BMI: Body mass index
CPRS: Computerized patient record system
ETR: End of treatment response
EVR: Early virologic response
HCC: Hepatocellular carcinoma
HCV: Hepatitis C virus
HIV: Human immunodeficiency virus
PEG-INF: Pegylated interferon
RVR: Rapid virologic response
SVR: Sustained virologic response
VA: Veterans affairs
VAMC: Veterans Affairs Medical Center
VL: Viral load.
